# The permeability and selectivity of nanocomposite membrane of PEBAx 1657/PEI/SiO_2_ for separation of CO_2_, N_2_, O_2_, CH_4_ gases: A data set

**DOI:** 10.1016/j.dib.2019.104800

**Published:** 2019-11-16

**Authors:** Mojtaba Shafiee, Ali Akbari, Iman Bahreini pour, Rauf Foroutan, Bahaman Ramavandi

**Affiliations:** a Department of Chemical Engineering, Jundi-Shapur University of Technology, P.O. Box 64615-334, Dezful, Iran; b Polymer Research Laboratory, Department of Organic and Biochemistry, Faculty of Chemistry, University of Tabriz, Tabriz, Iran; c Systems Environmental Health and Energy Research Center, The Persian Gulf Biomedical Sciences Research Institute, Bushehr University of Medical Sciences, Bushehr, Iran; d Department of Environmental Health Engineering, Faculty of Health and Nutrition, Bushehr University of Medical Sciences, Bushehr, Iran

**Keywords:** Membrane, Thermal fission, Poly ether-block-amide, Poly-ether-imide, Silica nanoparticles

## Abstract

The poly ether-block-amide (PEBAx)/Poly-ether-imide (PEI)/SiO_2_ nanocomposite membranes were fabricated using the solution casting method and utilized for separation of N_2_, O_2_, CH_4,_ and CO_2_ gases. The effect of SiO_2_ nanoparticles loading on permeability and selectivity of gases using the nanocomposite membranes was tested. The data showed that the permeability of the gases increased with increasing SiO_2_ nanoparticle content. dBy adding SiO_2_ nanoparticles (10 wt%), the permeability of N_2_, O_2_, CH_4,_ and CO_2_ gases elevated from 0.39, 1, 1.83 and 11.1 to 2.01, 1.95, 2.98 and 19.83 Barrer unit, respectively (at a pressure of 2 Bar). In contrast, with increasing SiO_2_ content the selectivity of the studied gases decreased. The morphology, crystallinity and the functional groups of the fabricated membranes were evaluated using scanning electron microscopy (SEM), X-ray diffraction (XRD) and Fourier-transform infrared spectroscopy (FTIR) techniques. The data presented confirm the influence of the nanoparticles on the membrane structure and thus on the permeability and selectivity of the membranes.

**Table:** Specification Table

Subject area	Chemical engineering
More subject area	Gas separation
Type of data	Figure, image, table
How data was acquired	- FTIR (Spectrum-65, Perkin Elmer) analysis was used to investigate the functional groups of membranes - XRD patterns of fabricated membranes were prepared using X-Ray Diffractometer, Bruker, D8-Advance model. - Scanning electron microscopy (MIRA3, Tescan) was utilized to study the morphology of prepared membranes
Data Format	Raw
Experimental factors	• The membranes were fabricated using the phase inversion method. • SiO_2_ nanoparticles were used to modify the permeability of membranes. • The permeability and selectivity of membranes were investigated. • The constant temperature- pressure method was used to measure the permeability of membranes.
Experimental features	Separation of N_2_, O_2_, CH_4_, and CO_2_ gases using Pebax/PEI- SiO_2_ nanocomposite membranes.
Data source location	Jundi Shapur University of Technology, Dezful, Iran
Data accessibility	Data were presented with the article.

**Table:** 

**Value of the Data** • This data can be useful for developing the nanocomposite polymeric membranes. • The study may be applicable for oil, gas and petrochemical industries for natural gas sweetening and purification processes. • Our data can be helpful for power plants to the separation of CO_2_ gas released from fossil fuels. • This paper introduces a membrane to the world of industry that can be useful for controlling carbon dioxide gas and thus controlling global warming.

## 1 Data

**Fig. 1:**
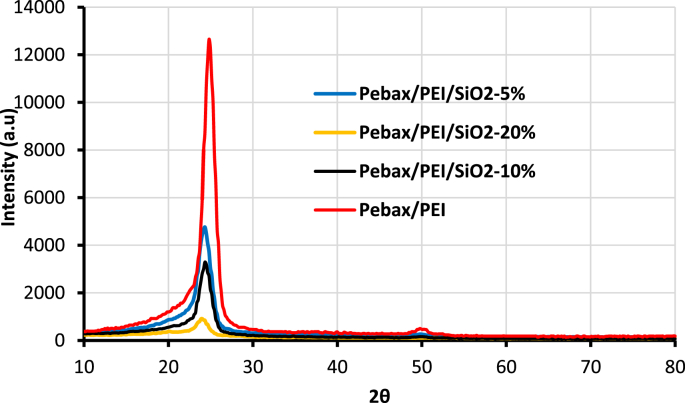
XRD pattern of fabricated membranes.

**Fig. 2:**
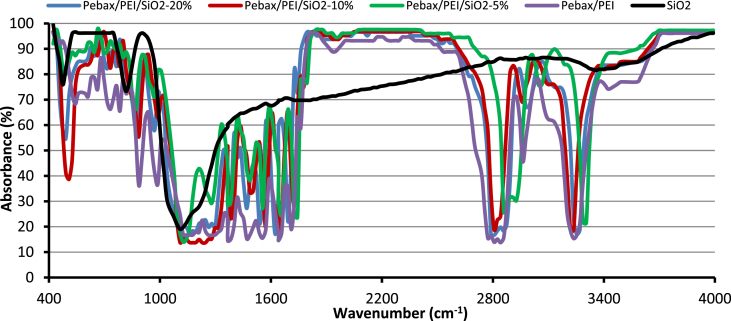
FTIR spectrum of prepared membranes.

**Fig. 3:**
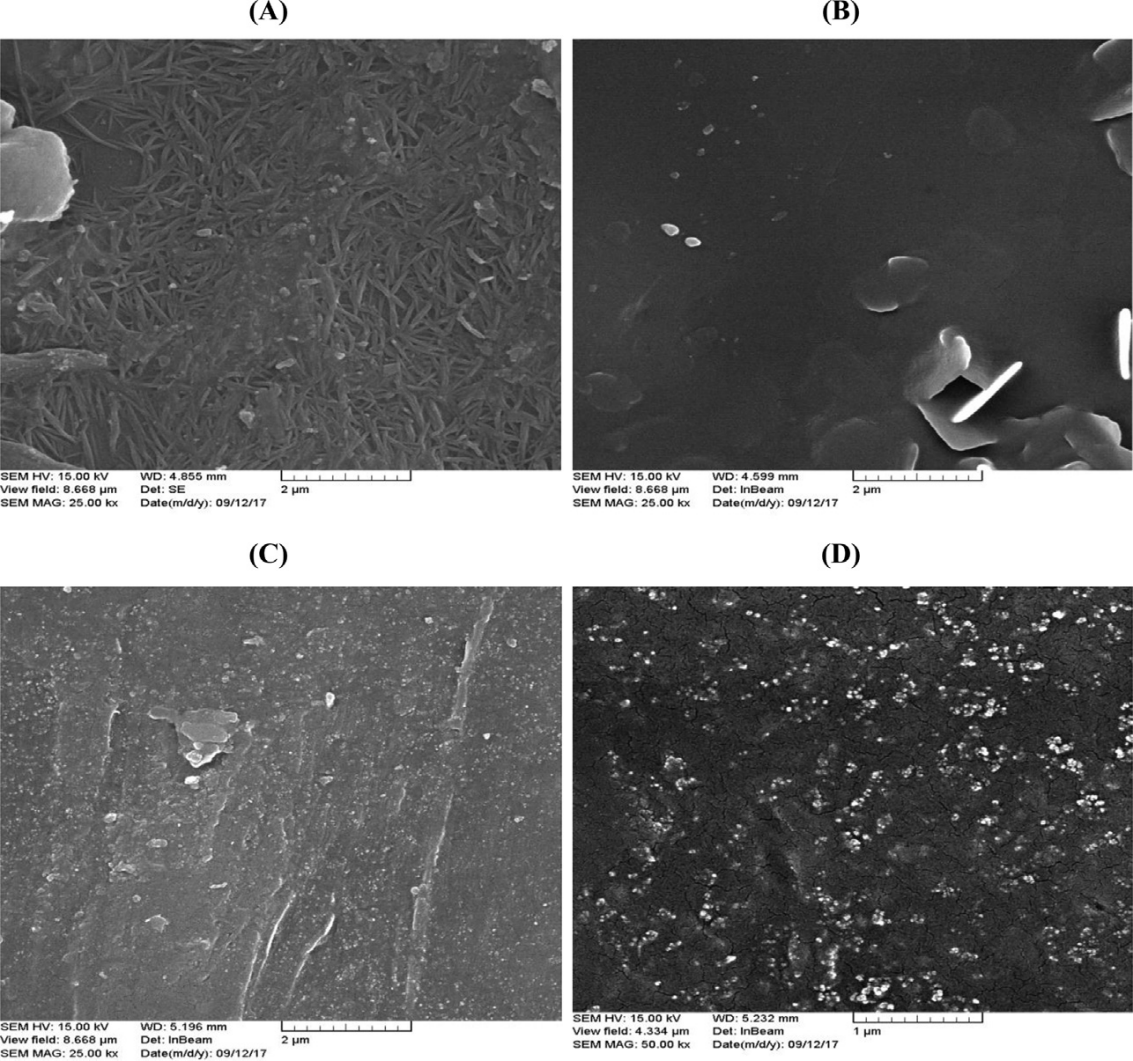
SEM images of fabricated membranes, **(A)** Pebax/PEI, **(B)** Pebax/PEI/$SiO_{2}$-5%, **(C)** Pebax/PEI/$SiO_{2}$-10% and **(D)** Pebax/PEI/$SiO_{2}$-20%.

**Fig. 4:**
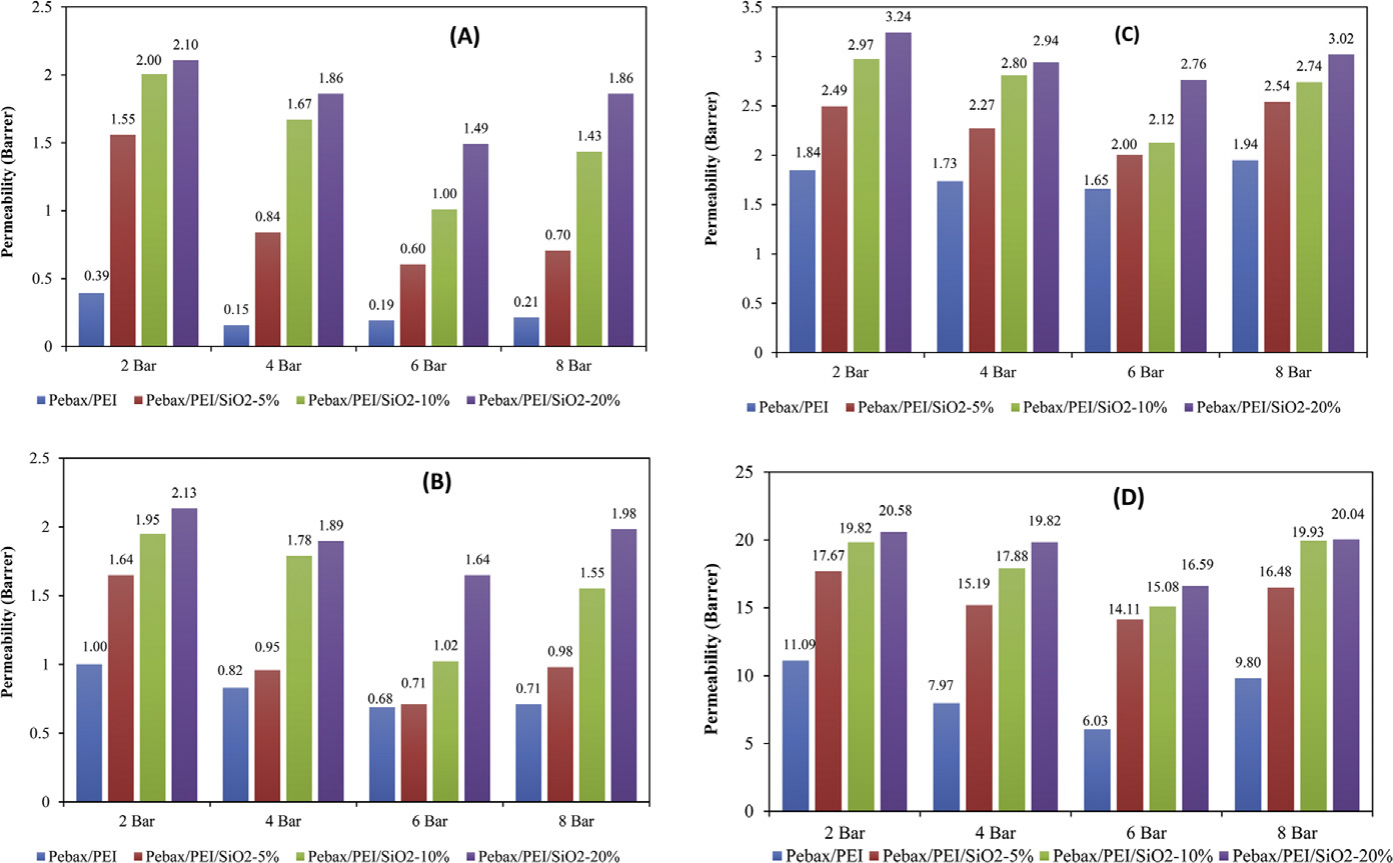
The effect of nanoparticle loading on gases permeability in various feed gas pressure, **(A)**$N_{2}$, **(B)**$O_{2}$, **(C)**$CH_{4}$, and **(D)**$CO_{2}$.

The X-Ray Diffraction (XRD) of SiO_2_ nanoparticles and fabricated membranes is shown in Fig. 1. Also, the FTIR analysis of SiO_2_ nanoparticles and membranes are depicted in Fig. 2. Four SEM images of prepared membranes have been indicated in Fig. 3. The effect of SiO_2_ nanoparticle loading on permeability in various feed gas pressure is shown in Fig. 4. Table 1 is related to the kinetic diameter and condensability of the studied gases. The selectivity of CH_4_, N_2_, and O_2_ gases is depicted in Table 2. The raw data for this work are presented in the Supplementary section.

**Table 1:** The kinetic diameter and condensability.

Gas	Kinetic diameter (Å)	Condensability (K)
${\text{Co}}_{2}$	3.30	195
${\text{O}}_{2}$	3.46	107
${\text{N}}_{2}$	3.64	71
${\text{CH}}_{4}$	3.80	149

**Table 2:** The selectivity of prepared membranes.

Pressure	Gas type	Membrane type			
		Pebax/PEI	Pebax/PEI-5%	Pebax/PEI-10%	Pebax/PEI-20%
2 Bar	${\text{CH}}_{4}$	6.05	7.08	6.66	6.34
	${\text{O}}_{2}$	11.07	10.71	10.16	9.64
	${\text{N}}_{2}$	28.28	11.34	9.88	9.76
4 Bar	${\text{CH}}_{4}$	4.59	6.68	6.37	6.73
	${\text{O}}_{2}$	9.61	15.84	10	10.45
	${\text{N}}_{2}$	50.80	18.07	10.70	10.65
6 Bar	${\text{CH}}_{4}$	3.63	7.04	7.09	6.00
	${\text{O}}_{2}$	8.74	19.84	14.73	10.06
	${\text{N}}_{2}$	31.66	23.31	14.95	11.12
8 Bar	${\text{CH}}_{4}$	5.03	6.49	7.27	6.63
	${\text{O}}_{2}$	13.78	16.81	12.84	10.10
	${\text{N}}_{2}$	46.03	23.34	13.89	10.77

## 2 Experimental design, materials, and methods

### 2.1 Membrane fabrication

**Fig. 5:**
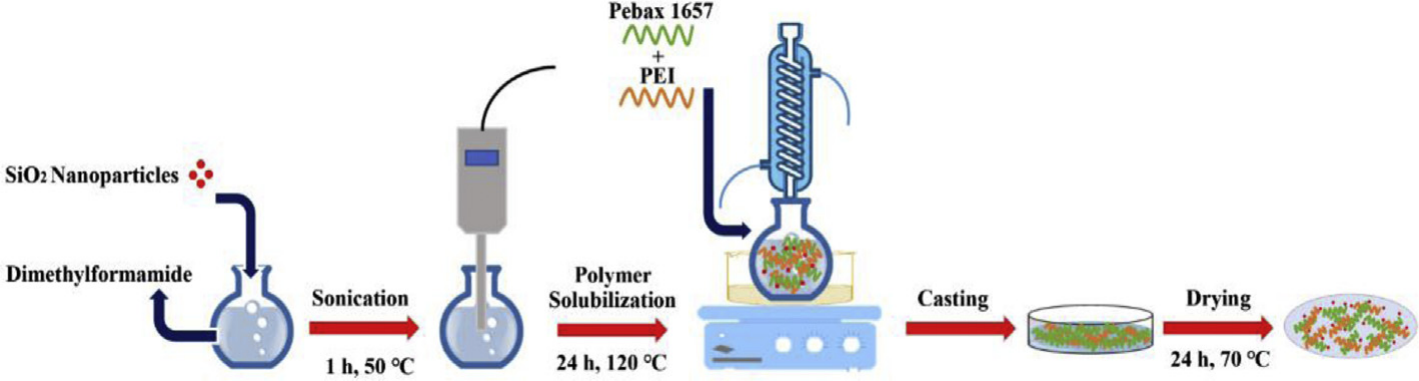
A schematic for membrane fabrication.

The polymer membranes were prepared by solution casting method [1]. To prepare the PEBA/PEI/$SiO_{2}$ nanocomposite membranes with 0, 5, 10 and 20 wt% of $SiO_{2}$ nanoparticles, first $SiO_{2}$ nanoparticles were dispersed in di-methyl-formamide by the ultrasonic process for 60 min at 50 °C. A 6% w/v of polymer solution was fabricated by adding Pebax 1657 and PEI (4:1 wt ratio) to the solution at 120 °C. To form a homogeneous solution, the mixing was continued for 24 h. Afterward, the solution was poured on Teflon mold at 70 °C. Then, the solvent completely removed from the membranes by vacuum drying in the ambient temperature for 4 h. Finally, the thickness of the prepared membranes was measured by a micrometer. A schematic for the membrane fabrication is illustrated in Fig. 5.

### 2.2 Gas permeability and selectivity measurement

The gas permeability was measured using the time lag method [2,3]. The gas flow rate was obtained using a constant pressure method. In this method, by connecting the downstream space to the water column, and by measuring the changes of water column's height over time, the gas flow rate passing through the membrane is obtained. The gas flow rate can achieve from the slope of the linear part of the water column height as a function of time. Then, the permeability coefficient can be calculated using the following equation [4]:

(1) $$P=\frac{Q\;.\;l}{\left(\;P_{1}-\;P_{2}\;\right)\;.\;A}$$

where P is the gas permeability coefficient in the polymer (1 Barrer = $cm^{3}$ (STP).cm/$cm^{2}$.S.cmHg), Q is the gas flow rate ($cm^{3}$/s), l is the membrane thickness (cm), A is the cross-section area of membrane ($cm^{2}$), $P_{1}$ and $\;P_{2}$ are the gas pressure in upstream and downstream, respectively. The membrane ideal selectivity respect to a given gas can calculate through the following equation [5]:

(2) $$\alpha =P_{i}/P_{j}\;$$

where α is the membrane ideal selectivity, $P_{i}$ and $P_{j}$ are the gas permeability coefficient of gas (i) and (j), respectively.
