# Supplementary material for: The permeability and selectivity of nanocomposite membrane of PEBAx 1657/PEI/SiO2 for separation of CO2, N2, O2, CH4 gases: A data set
Source: Data Brief. 2019 Nov 16;28:104800. doi: 10.1016/j.dib.2019.104800 (PMC6889794; doi:10.1016/j.dib.2019.104800)
Supplement: Multimedia component 1 [file mmc1.docx]

**Data source for**

**The permeability and selectivity of nanocomposite membrane of PEBAx 1657/PEI/SiO_2_ for separation of CO_2_, N_2_, O_2_, CH_4_ gases: A data set**

Mojtaba Shafiee^1^, Ali Akbari^1^ Iman Bahreini pour^1^, Rauf Foroutan^2^*, Bahaman Ramavandi^3,4^*

^1^ Department of Chemical Engineering, Jundi-Shapur University of Technology, P.O. Box 64615-334, Dezful, Iran

^2^ Polymer Research Laboratory, Department of Organic and Biochemistry, Faculty of Chemistry, University of Tabriz, Tabriz, Iran

^3^ Systems Environmental Health and Energy Research Center, The Persian Gulf Biomedical Sciences Research Institute, Bushehr University of Medical Sciences, Bushehr,

Iran

^4^ Department of Environmental Health Engineering, Faculty of Health and Nutrition, Bushehr University of Medical Sciences, Bushehr, Iran

***Corresponding authors:** R. Foroutan (email: rauf.foroutan@yahoo.com; phone: +989174015168) and B. Ramavandi (email: [b.ramavandi@bpums.ac.ir](mailto:b.ramavandi@bpums.ac.ir), phone: +989363311903)

**Table S1**. Data source for Fig. 1 (XRD pattern of fabricated membranes).

| 2θ | Pebax/PEI/SiO2-5% | Pebax/PEI/SiO2-20% | Pebax/PEI/SiO2-10% | Pebax/PEI |
| --- | --- | --- | --- | --- |
| 9.76593 | 352.06 | 247.191 | 287.381 | 388.548 |
| 9.92 | 348.315 | 243.446 | 289.272 | 398.773 |
| 10.0859 | 335.206 | 230.337 | 277.928 | 368.098 |
| 10.24 | 340.824 | 228.464 | 279.819 | 378.323 |
| 10.4059 | 331.461 | 228.464 | 285.491 | 398.773 |
| 10.56 | 337.079 | 239.7 | 285.491 | 388.548 |
| 10.7259 | 325.843 | 226.592 | 285.491 | 388.548 |
| 10.88 | 340.824 | 220.974 | 296.835 | 388.548 |
| 11.0459 | 344.569 | 220.974 | 298.726 | 388.548 |
| 11.2 | 346.442 | 224.719 | 296.835 | 388.548 |
| 11.3659 | 353.933 | 230.337 | 291.163 | 398.773 |
| 11.52 | 359.551 | 237.828 | 291.163 | 398.773 |
| 11.6859 | 361.423 | 228.464 | 298.726 | 429.448 |
| 11.84 | 359.551 | 222.846 | 296.835 | 408.998 |
| 12.0059 | 344.569 | 226.592 | 291.163 | 439.673 |
| 12.16 | 346.442 | 220.974 | 291.163 | 460.123 |
| 12.3259 | 357.678 | 224.719 | 293.053 | 460.123 |
| 12.48 | 363.296 | 224.719 | 302.507 | 501.022 |
| 12.6459 | 361.423 | 235.955 | 304.398 | 460.123 |
| 12.8 | 361.423 | 226.592 | 311.96 | 521.472 |
| 12.9659 | 368.914 | 219.101 | 302.507 | 460.123 |
| 13.12 | 376.404 | 219.101 | 293.053 | 501.022 |
| 13.2859 | 385.768 | 228.464 | 310.07 | 511.247 |
| 13.44 | 382.022 | 234.082 | 313.851 | 501.022 |
| 13.6059 | 389.513 | 239.7 | 317.632 | 480.573 |
| 13.76 | 389.513 | 234.082 | 317.632 | 541.922 |
| 13.9259 | 393.258 | 232.21 | 315.742 | 511.247 |
| 14.08 | 397.004 | 235.955 | 308.179 | 521.472 |
| 14.2459 | 400.749 | 235.955 | 323.304 | 511.247 |
| 14.4 | 400.749 | 232.21 | 323.304 | 552.147 |
| 14.5659 | 413.858 | 245.318 | 325.195 | 552.147 |
| 14.72 | 417.603 | 252.809 | 327.086 | 552.147 |
| 14.8859 | 428.839 | 245.318 | 338.43 | 593.047 |
| 15.04 | 430.712 | 245.318 | 338.43 | 603.272 |
| 15.2059 | 449.438 | 262.172 | 357.336 | 603.272 |
| 15.36 | 458.801 | 264.045 | 368.68 | 644.172 |
| 15.5259 | 479.401 | 262.172 | 378.134 | 644.172 |
| 15.68 | 486.891 | 264.045 | 378.134 | 644.172 |
| 15.8459 | 496.255 | 267.79 | 383.806 | 644.172 |
| 16 | 494.382 | 267.79 | 374.352 | 685.072 |
| 16.1659 | 505.618 | 265.918 | 378.134 | 695.297 |
| 16.32 | 516.854 | 265.918 | 378.134 | 674.847 |
| 16.4859 | 526.217 | 265.918 | 383.806 | 736.196 |
| 16.64 | 537.453 | 262.172 | 398.931 | 736.196 |
| 16.8059 | 541.199 | 265.918 | 400.822 | 736.196 |
| 16.96 | 535.581 | 262.172 | 395.15 | 766.871 |
| 17.1259 | 552.434 | 275.281 | 400.822 | 777.096 |
| 17.28 | 548.689 | 277.154 | 410.275 | 746.421 |
| 17.4459 | 552.434 | 282.772 | 423.51 | 787.321 |
| 17.6 | 584.27 | 277.154 | 425.4 | 828.221 |
| 17.7659 | 606.742 | 279.026 | 432.963 | 838.446 |
| 17.92 | 616.105 | 286.517 | 446.198 | 920.245 |
| 18.0859 | 629.213 | 297.753 | 459.432 | 920.245 |
| 18.24 | 625.468 | 307.116 | 449.979 | 920.245 |
| 18.4059 | 672.285 | 297.753 | 457.542 | 920.245 |
| 18.56 | 691.011 | 295.88 | 455.651 | 920.245 |
| 18.7259 | 694.757 | 312.734 | 470.776 | 950.92 |
| 18.88 | 691.011 | 320.225 | 484.011 | 1002.04 |
| 19.0459 | 705.993 | 312.734 | 489.683 | 1012.27 |
| 19.2 | 730.337 | 329.588 | 487.792 | 1053.17 |
| 19.3659 | 764.045 | 342.697 | 502.918 | 1094.07 |
| 19.52 | 779.026 | 344.569 | 510.48 | 1104.29 |
| 19.6859 | 810.861 | 344.569 | 527.496 | 1134.97 |
| 19.84 | 842.697 | 355.805 | 544.512 | 1196.32 |
| 20.0059 | 868.914 | 378.277 | 555.856 | 1196.32 |
| 20.16 | 876.404 | 380.15 | 569.091 | 1216.77 |
| 20.3259 | 882.022 | 363.296 | 587.998 | 1257.67 |
| 20.48 | 891.386 | 353.933 | 605.014 | 1359.92 |
| 20.6459 | 926.966 | 353.933 | 623.92 | 1380.37 |
| 20.8 | 934.457 | 353.933 | 620.139 | 1359.92 |
| 20.9659 | 979.401 | 353.933 | 620.139 | 1400.82 |
| 21.12 | 994.382 | 350.187 | 622.03 | 1472.39 |
| 21.2859 | 1044.94 | 359.551 | 625.811 | 1472.39 |
| 21.44 | 1052.43 | 361.423 | 659.843 | 1564.42 |
| 21.6059 | 1104.87 | 372.659 | 697.656 | 1564.42 |
| 21.76 | 1129.21 | 380.15 | 697.656 | 1615.54 |
| 21.9259 | 1166.67 | 393.258 | 722.235 | 1697.34 |
| 22.08 | 1250.94 | 395.131 | 748.704 | 1809.82 |
| 22.2459 | 1280.9 | 404.494 | 828.112 | 1922.29 |
| 22.4 | 1331.46 | 400.749 | 847.019 | 2014.31 |
| 22.5659 | 1440.07 | 438.202 | 899.958 | 2116.56 |
| 22.8859 | 1634.83 | 494.382 | 949.115 | 2259.71 |
| 23.04 | 1827.72 | 529.963 | 1085.24 | 2341.51 |
| 23.2059 | 2112.36 | 591.76 | 1166.54 | 2668.71 |
| 23.36 | 2689.14 | 689.139 | 1573.04 | 2862.99 |
| 23.5259 | 2951.31 | 769.663 | 1712.94 | 3435.58 |
| 23.68 | 3627.34 | 795.88 | 2134.56 | 3752.56 |
| 23.8459 | 4117.98 | 900.749 | 2488.12 | 4877.3 |
| 24 | 4441.95 | 910.112 | 2813.31 | 5930.47 |
| 24.1659 | 4631.09 | 857.678 | 3008.05 | 7995.91 |
| 24.32 | 4760.3 | 840.824 | 3289.76 | 8752.56 |
| 24.4859 | 4481.27 | 696.629 | 3195.23 | 10715.7 |
| 24.64 | 4080.52 | 672.285 | 2851.13 | 11758.7 |
| 24.8059 | 3734.08 | 539.326 | 2705.55 | 12648.3 |
| 24.96 | 3059.93 | 460.674 | 2155.36 | 12290.4 |
| 25.1259 | 2743.45 | 391.386 | 1949.28 | 10961.1 |
| 25.28 | 2104.87 | 361.423 | 1455.81 | 10020.4 |
| 25.4459 | 1752.81 | 299.625 | 1261.08 | 7801.64 |
| 25.6 | 1269.66 | 286.517 | 892.395 | 6687.12 |
| 25.7659 | 1091.76 | 245.318 | 771.392 | 4662.58 |
| 25.92 | 788.39 | 232.21 | 572.872 | 4069.53 |
| 26.0859 | 720.974 | 226.592 | 495.355 | 2648.26 |
| 26.24 | 631.086 | 220.974 | 408.384 | 2085.89 |
| 26.4059 | 593.633 | 209.738 | 391.368 | 1472.39 |
| 26.56 | 529.963 | 217.228 | 357.336 | 1298.57 |
| 26.7259 | 501.873 | 213.483 | 357.336 | 1053.17 |
| 26.88 | 496.255 | 217.228 | 336.539 | 981.595 |
| 27.0459 | 488.764 | 209.738 | 332.758 | 879.346 |
| 27.2 | 479.401 | 198.502 | 298.726 | 828.221 |
| 27.3659 | 453.184 | 187.266 | 291.163 | 756.646 |
| 27.52 | 423.221 | 194.757 | 281.709 | 736.196 |
| 27.6859 | 398.876 | 191.011 | 285.491 | 674.847 |
| 27.84 | 385.768 | 192.884 | 276.037 | 644.172 |
| 28.0059 | 383.895 | 187.266 | 257.131 | 633.947 |
| 28.16 | 370.787 | 192.884 | 257.131 | 593.047 |
| 28.3259 | 368.914 | 192.884 | 255.24 | 552.147 |
| 28.48 | 365.169 | 192.884 | 257.131 | 552.147 |
| 28.6459 | 359.551 | 185.393 | 245.787 | 552.147 |
| 28.8 | 344.569 | 185.393 | 242.005 | 552.147 |
| 28.9659 | 350.187 | 170.412 | 243.896 | 552.147 |
| 29.12 | 355.805 | 168.539 | 243.896 | 531.697 |
| 29.2859 | 346.442 | 162.921 | 213.645 | 511.247 |
| 29.44 | 327.715 | 161.049 | 209.864 | 490.798 |
| 29.6059 | 318.352 | 159.176 | 215.536 | 460.123 |
| 29.76 | 308.989 | 157.303 | 207.973 | 470.348 |
| 29.9259 | 305.243 | 155.431 | 215.536 | 460.123 |
| 30.08 | 305.243 | 153.558 | 215.536 | 460.123 |
| 30.2459 | 312.734 | 144.195 | 200.411 | 460.123 |
| 30.4 | 314.607 | 142.322 | 200.411 | 460.123 |
| 30.5659 | 305.243 | 134.831 | 200.411 | 429.448 |
| 30.72 | 282.772 | 134.831 | 190.957 | 408.998 |
| 30.8859 | 286.517 | 142.322 | 198.52 | 419.223 |
| 31.04 | 288.39 | 136.704 | 196.629 | 419.223 |
| 31.2059 | 284.644 | 140.449 | 198.52 | 378.323 |
| 31.36 | 288.39 | 142.322 | 198.52 | 368.098 |
| 31.5259 | 280.899 | 144.195 | 192.848 | 368.098 |
| 31.68 | 282.772 | 131.086 | 190.957 | 368.098 |
| 31.8459 | 282.772 | 140.449 | 177.723 | 388.548 |
| 32 | 277.154 | 138.577 | 175.832 | 368.098 |
| 32.1659 | 279.026 | 136.704 | 179.613 | 368.098 |
| 32.32 | 271.536 | 138.577 | 177.723 | 368.098 |
| 32.4859 | 280.899 | 146.067 | 173.941 | 368.098 |
| 32.64 | 290.262 | 149.813 | 173.941 | 368.098 |
| 32.8059 | 280.899 | 147.94 | 166.379 | 368.098 |
| 32.96 | 265.918 | 147.94 | 166.379 | 368.098 |
| 33.1259 | 269.663 | 149.813 | 173.941 | 368.098 |
| 33.28 | 269.663 | 144.195 | 172.051 | 368.098 |
| 33.4459 | 260.3 | 136.704 | 164.488 | 368.098 |
| 33.6 | 269.663 | 125.468 | 172.051 | 357.873 |
| 33.7659 | 271.536 | 125.468 | 172.051 | 368.098 |
| 33.92 | 260.3 | 127.341 | 170.16 | 368.098 |
| 34.0859 | 256.554 | 125.468 | 158.816 | 327.198 |
| 34.24 | 267.79 | 125.468 | 158.816 | 357.873 |
| 34.4059 | 264.045 | 125.468 | 156.925 | 357.873 |
| 34.56 | 260.3 | 127.341 | 168.269 | 327.198 |
| 34.7259 | 265.918 | 131.086 | 183.395 | 327.198 |
| 34.88 | 271.536 | 132.959 | 181.504 | 327.198 |
| 35.0459 | 264.045 | 138.577 | 156.925 | 327.198 |
| 35.2 | 262.172 | 140.449 | 158.816 | 327.198 |
| 35.3659 | 264.045 | 129.213 | 156.925 | 368.098 |
| 35.52 | 247.191 | 129.213 | 158.816 | 347.648 |
| 35.6859 | 247.191 | 134.831 | 156.925 | 357.873 |
| 35.84 | 249.064 | 131.086 | 156.925 | 368.098 |
| 36.0059 | 247.191 | 129.213 | 156.925 | 368.098 |
| 36.16 | 241.573 | 125.468 | 147.472 | 327.198 |
| 36.3259 | 247.191 | 123.596 | 149.363 | 357.873 |
| 36.48 | 250.936 | 127.341 | 158.816 | 368.098 |
| 36.6459 | 245.318 | 125.468 | 156.925 | 327.198 |
| 36.8 | 250.936 | 125.468 | 155.035 | 347.648 |
| 36.9659 | 243.446 | 123.596 | 155.035 | 368.098 |
| 37.12 | 239.7 | 123.596 | 147.472 | 357.873 |
| 37.2859 | 245.318 | 123.596 | 147.472 | 368.098 |
| 37.44 | 245.318 | 123.596 | 143.691 | 368.098 |
| 37.6059 | 247.191 | 123.596 | 141.8 | 368.098 |
| 37.76 | 239.7 | 125.468 | 141.8 | 368.098 |
| 37.9259 | 247.191 | 125.468 | 141.8 | 327.198 |
| 38.08 | 245.318 | 125.468 | 145.581 | 327.198 |
| 38.2459 | 234.082 | 125.468 | 147.472 | 306.748 |
| 38.4 | 237.828 | 117.978 | 143.691 | 337.423 |
| 38.5659 | 237.828 | 117.978 | 136.128 | 368.098 |
| 38.72 | 234.082 | 117.978 | 136.128 | 347.648 |
| 38.8859 | 237.828 | 125.468 | 134.237 | 337.423 |
| 39.04 | 237.828 | 125.468 | 134.237 | 327.198 |
| 39.2059 | 245.318 | 114.232 | 139.909 | 327.198 |
| 39.36 | 245.318 | 114.232 | 139.909 | 316.973 |
| 39.5259 | 235.955 | 117.978 | 128.565 | 296.524 |
| 39.68 | 217.228 | 117.978 | 136.128 | 296.524 |
| 39.8459 | 224.719 | 121.723 | 130.456 | 347.648 |
| 40 | 235.955 | 119.85 | 136.128 | 357.873 |
| 40.1659 | 237.828 | 108.614 | 149.363 | 296.524 |
| 40.32 | 228.464 | 108.614 | 138.019 | 296.524 |
| 40.4859 | 228.464 | 110.487 | 136.128 | 286.299 |
| 40.64 | 220.974 | 108.614 | 139.909 | 296.524 |
| 40.8059 | 224.719 | 108.614 | 139.909 | 347.648 |
| 40.96 | 222.846 | 108.614 | 136.128 | 327.198 |
| 41.1259 | 220.974 | 108.614 | 126.675 | 306.748 |
| 41.28 | 211.61 | 110.487 | 130.456 | 296.524 |
| 41.4459 | 213.483 | 108.614 | 130.456 | 316.973 |
| 41.6 | 222.846 | 108.614 | 134.237 | 327.198 |
| 41.7659 | 226.592 | 110.487 | 143.691 | 296.524 |
| 41.92 | 228.464 | 108.614 | 143.691 | 296.524 |
| 42.0859 | 215.356 | 108.614 | 136.128 | 296.524 |
| 42.24 | 219.101 | 108.614 | 138.019 | 286.299 |
| 42.4059 | 226.592 | 108.614 | 138.019 | 286.299 |
| 42.56 | 217.228 | 108.614 | 134.237 | 286.299 |
| 42.7259 | 209.738 | 108.614 | 134.237 | 276.074 |
| 42.88 | 213.483 | 108.614 | 134.237 | 276.074 |
| 43.0459 | 213.483 | 108.614 | 126.675 | 296.524 |
| 43.2 | 215.356 | 117.978 | 124.784 | 296.524 |
| 43.3659 | 215.356 | 117.978 | 122.893 | 276.074 |
| 43.52 | 213.483 | 116.105 | 132.347 | 286.299 |
| 43.6859 | 213.483 | 110.487 | 122.893 | 296.524 |
| 43.84 | 220.974 | 110.487 | 122.893 | 276.074 |
| 44.0059 | 220.974 | 108.614 | 124.784 | 276.074 |
| 44.16 | 215.356 | 110.487 | 124.784 | 286.299 |
| 44.3259 | 213.483 | 119.85 | 124.784 | 276.074 |
| 44.48 | 219.101 | 117.978 | 124.784 | 276.074 |
| 44.6459 | 219.101 | 108.614 | 132.347 | 296.524 |
| 44.8 | 217.228 | 108.614 | 132.347 | 276.074 |
| 44.9659 | 217.228 | 108.614 | 132.347 | 286.299 |
| 45.12 | 232.21 | 108.614 | 132.347 | 276.074 |
| 45.2859 | 234.082 | 108.614 | 132.347 | 306.748 |
| 45.44 | 219.101 | 108.614 | 134.237 | 276.074 |
| 45.6059 | 215.356 | 110.487 | 134.237 | 276.074 |
| 45.76 | 217.228 | 112.36 | 134.237 | 276.074 |
| 45.9259 | 226.592 | 108.614 | 132.347 | 276.074 |
| 46.08 | 222.846 | 108.614 | 132.347 | 276.074 |
| 46.2459 | 224.719 | 110.487 | 122.893 | 276.074 |
| 46.4 | 219.101 | 108.614 | 124.784 | 296.524 |
| 46.5659 | 207.865 | 110.487 | 124.784 | 276.074 |
| 46.72 | 207.865 | 117.978 | 115.331 | 276.074 |
| 46.8859 | 207.865 | 108.614 | 122.893 | 276.074 |
| 47.04 | 204.12 | 110.487 | 122.893 | 276.074 |
| 47.2059 | 207.865 | 108.614 | 113.44 | 296.524 |
| 47.36 | 217.228 | 108.614 | 113.44 | 276.074 |
| 47.5259 | 215.356 | 108.614 | 122.893 | 286.299 |
| 47.68 | 205.993 | 108.614 | 122.893 | 276.074 |
| 47.8459 | 200.375 | 108.614 | 122.893 | 276.074 |
| 48 | 204.12 | 108.614 | 122.893 | 286.299 |
| 48.1659 | 204.12 | 108.614 | 124.784 | 286.299 |
| 48.32 | 211.61 | 108.614 | 124.784 | 286.299 |
| 48.4859 | 207.865 | 104.869 | 124.784 | 337.423 |
| 48.64 | 215.356 | 101.124 | 124.784 | 316.973 |
| 48.8059 | 222.846 | 101.124 | 138.019 | 368.098 |
| 48.96 | 234.082 | 101.124 | 139.909 | 368.098 |
| 49.1259 | 237.828 | 99.2509 | 143.691 | 368.098 |
| 49.28 | 237.828 | 99.2509 | 141.8 | 388.548 |
| 49.4459 | 243.446 | 106.742 | 147.472 | 460.123 |
| 49.6 | 252.809 | 104.869 | 153.144 | 460.123 |
| 49.7659 | 264.045 | 106.742 | 156.925 | 501.022 |
| 49.92 | 262.172 | 102.996 | 156.925 | 480.573 |
| 50.0859 | 254.682 | 106.742 | 156.925 | 460.123 |
| 50.24 | 247.191 | 106.742 | 153.144 | 470.348 |
| 50.4059 | 237.828 | 110.487 | 147.472 | 460.123 |
| 50.56 | 219.101 | 108.614 | 145.581 | 449.898 |
| 50.7259 | 213.483 | 106.742 | 126.675 | 368.098 |
| 50.88 | 209.738 | 101.124 | 124.784 | 327.198 |
| 51.0459 | 207.865 | 101.124 | 113.44 | 316.973 |
| 51.2 | 205.993 | 104.869 | 121.003 | 296.524 |
| 51.3659 | 204.12 | 108.614 | 109.659 | 276.074 |
| 51.52 | 194.757 | 108.614 | 107.768 | 276.074 |
| 51.6859 | 187.266 | 110.487 | 113.44 | 276.074 |
| 51.84 | 196.629 | 108.614 | 113.44 | 265.849 |
| 52.0059 | 198.502 | 95.5056 | 105.877 | 276.074 |
| 52.16 | 185.393 | 97.3783 | 105.877 | 276.074 |
| 52.3259 | 181.648 | 106.742 | 111.549 | 276.074 |
| 52.48 | 183.521 | 106.742 | 113.44 | 276.074 |
| 52.6459 | 181.648 | 104.869 | 111.549 | 245.399 |
| 52.8 | 177.903 | 104.869 | 107.768 | 235.174 |
| 52.9659 | 176.03 | 99.2509 | 107.768 | 235.174 |
| 53.12 | 181.648 | 99.2509 | 98.3147 | 235.174 |
| 53.2859 | 183.521 | 101.124 | 94.5334 | 224.949 |
| 53.44 | 174.157 | 95.5056 | 94.5334 | 214.724 |
| 53.6059 | 174.157 | 101.124 | 98.3147 | 204.499 |
| 53.76 | 181.648 | 101.124 | 98.3147 | 204.499 |
| 53.9259 | 183.521 | 104.869 | 103.987 | 184.049 |
| 54.08 | 172.285 | 102.996 | 115.331 | 194.274 |
| 54.2459 | 172.285 | 93.633 | 103.987 | 204.499 |
| 54.4 | 174.157 | 91.7603 | 81.2987 | 194.274 |
| 54.5659 | 172.285 | 91.7603 | 86.9707 | 204.499 |
| 54.72 | 170.412 | 99.2509 | 90.7521 | 214.724 |
| 54.8859 | 172.285 | 101.124 | 98.3147 | 184.049 |
| 55.04 | 168.539 | 91.7603 | 100.205 | 184.049 |
| 55.2059 | 170.412 | 93.633 | 94.5334 | 214.724 |
| 55.36 | 162.921 | 91.7603 | 94.5334 | 204.499 |
| 55.5259 | 162.921 | 91.7603 | 94.5334 | 194.274 |
| 55.68 | 161.049 | 89.8876 | 86.9707 | 184.049 |
| 55.8459 | 166.667 | 89.8876 | 90.7521 | 194.274 |
| 56 | 172.285 | 91.7603 | 88.8614 | 184.049 |
| 56.1659 | 168.539 | 93.633 | 98.3147 | 194.274 |
| 56.32 | 170.412 | 91.7603 | 90.7521 | 184.049 |
| 56.4859 | 168.539 | 91.7603 | 79.408 | 214.724 |
| 56.64 | 164.794 | 91.7603 | 88.8614 | 184.049 |
| 56.8059 | 168.539 | 91.7603 | 86.9707 | 184.049 |
| 56.96 | 172.285 | 91.7603 | 96.4241 | 184.049 |
| 57.1259 | 170.412 | 91.7603 | 98.3147 | 194.274 |
| 57.28 | 170.412 | 91.7603 | 102.096 | 184.049 |
| 57.4459 | 172.285 | 93.633 | 90.7521 | 184.049 |
| 57.6 | 159.176 | 91.7603 | 88.8614 | 184.049 |
| 57.7659 | 166.667 | 93.633 | 85.08 | 184.049 |
| 57.92 | 164.794 | 91.7603 | 86.9707 | 184.049 |
| 58.0859 | 151.685 | 86.1423 | 88.8614 | 184.049 |
| 58.24 | 151.685 | 84.2697 | 88.8614 | 184.049 |
| 58.4059 | 147.94 | 76.779 | 83.1894 | 184.049 |
| 58.56 | 153.558 | 84.2697 | 79.408 | 184.049 |
| 58.7259 | 157.303 | 88.015 | 77.5174 | 184.049 |
| 58.88 | 159.176 | 88.015 | 86.9707 | 184.049 |
| 59.0459 | 157.303 | 89.8876 | 86.9707 | 184.049 |
| 59.2 | 151.685 | 89.8876 | 88.8614 | 184.049 |
| 59.3659 | 157.303 | 84.2697 | 90.7521 | 184.049 |
| 59.52 | 155.431 | 84.2697 | 90.7521 | 184.049 |
| 59.6859 | 157.303 | 88.015 | 88.8614 | 194.274 |
| 59.84 | 161.049 | 88.015 | 86.9707 | 184.049 |
| 60.0059 | 162.921 | 84.2697 | 86.9707 | 184.049 |
| 60.16 | 155.431 | 86.1423 | 88.8614 | 184.049 |
| 60.3259 | 157.303 | 82.397 | 86.9707 | 184.049 |
| 60.48 | 155.431 | 76.779 | 86.9707 | 184.049 |
| 60.6459 | 149.813 | 84.2697 | 85.08 | 184.049 |
| 60.8 | 153.558 | 86.1423 | 86.9707 | 184.049 |
| 60.9659 | 151.685 | 84.2697 | 85.08 | 184.049 |
| 61.12 | 161.049 | 84.2697 | 83.1894 | 184.049 |
| 61.2859 | 159.176 | 84.2697 | 86.9707 | 184.049 |
| 61.44 | 157.303 | 93.633 | 86.9707 | 184.049 |
| 61.6059 | 155.431 | 86.1423 | 85.08 | 184.049 |
| 61.76 | 157.303 | 88.015 | 86.9707 | 184.049 |
| 61.9259 | 155.431 | 86.1423 | 85.08 | 184.049 |
| 62.08 | 151.685 | 86.1423 | 85.08 | 184.049 |
| 62.2459 | 147.94 | 86.1423 | 83.1894 | 184.049 |
| 62.4 | 151.685 | 84.2697 | 83.1894 | 184.049 |
| 62.5659 | 155.431 | 80.5243 | 79.408 | 184.049 |
| 62.72 | 159.176 | 84.2697 | 77.5174 | 184.049 |
| 62.8859 | 159.176 | 84.2697 | 83.1894 | 184.049 |
| 63.04 | 147.94 | 84.2697 | 85.08 | 184.049 |
| 63.2059 | 151.685 | 84.2697 | 86.9707 | 184.049 |
| 63.36 | 153.558 | 84.2697 | 85.08 | 184.049 |
| 63.5259 | 157.303 | 84.2697 | 85.08 | 184.049 |
| 63.68 | 161.049 | 84.2697 | 85.08 | 143.149 |
| 63.8459 | 155.431 | 76.779 | 83.1894 | 143.149 |
| 64 | 157.303 | 74.9064 | 77.5174 | 173.824 |
| 64.1659 | 159.176 | 76.779 | 77.5174 | 173.824 |
| 64.32 | 157.303 | 78.6517 | 77.5174 | 184.049 |
| 64.4859 | 151.685 | 84.2697 | 81.2987 | 184.049 |
| 64.64 | 149.813 | 84.2697 | 85.08 | 163.599 |
| 64.8059 | 155.431 | 84.2697 | 81.2987 | 163.599 |
| 64.96 | 159.176 | 86.1423 | 83.1894 | 153.374 |
| 65.1259 | 155.431 | 88.015 | 86.9707 | 153.374 |
| 65.28 | 151.685 | 86.1423 | 88.8614 | 184.049 |
| 65.4459 | 146.067 | 84.2697 | 77.5174 | 184.049 |
| 65.6 | 147.94 | 84.2697 | 71.8454 | 184.049 |
| 65.7659 | 147.94 | 76.779 | 68.064 | 153.374 |
| 65.92 | 153.558 | 78.6517 | 77.5174 | 153.374 |
| 66.0859 | 147.94 | 84.2697 | 71.8454 | 184.049 |
| 66.24 | 144.195 | 78.6517 | 75.6267 | 173.824 |
| 66.4059 | 144.195 | 74.9064 | 71.8454 | 163.599 |
| 66.56 | 151.685 | 84.2697 | 71.8454 | 184.049 |
| 66.7259 | 151.685 | 80.5243 | 75.6267 | 143.149 |
| 66.88 | 151.685 | 74.9064 | 71.8454 | 184.049 |
| 67.0459 | 147.94 | 76.779 | 66.1734 | 132.924 |
| 67.2 | 147.94 | 76.779 | 79.408 | 143.149 |
| 67.3659 | 147.94 | 76.779 | 79.408 | 143.149 |
| 67.52 | 147.94 | 74.9064 | 79.408 | 163.599 |
| 67.6859 | 147.94 | 84.2697 | 79.408 | 153.374 |
| 67.84 | 151.685 | 84.2697 | 79.408 | 143.149 |
| 68.0059 | 153.558 | 84.2697 | 79.408 | 143.149 |
| 68.16 | 146.067 | 84.2697 | 71.8454 | 153.374 |
| 68.3259 | 146.067 | 84.2697 | 71.8454 | 184.049 |
| 68.48 | 149.813 | 84.2697 | 81.2987 | 184.049 |
| 68.6459 | 149.813 | 84.2697 | 68.064 | 163.599 |
| 68.8 | 147.94 | 82.397 | 69.9547 | 143.149 |
| 68.9659 | 149.813 | 84.2697 | 68.064 | 143.149 |
| 69.12 | 151.685 | 84.2697 | 77.5174 | 184.049 |
| 69.2859 | 146.067 | 84.2697 | 83.1894 | 184.049 |
| 69.44 | 147.94 | 84.2697 | 83.1894 | 153.374 |
| 69.6059 | 140.449 | 84.2697 | 69.9547 | 153.374 |
| 69.76 | 140.449 | 84.2697 | 71.8454 | 153.374 |
| 69.9259 | 146.067 | 84.2697 | 73.736 | 143.149 |
| 70.08 | 147.94 | 84.2697 | 73.736 | 173.824 |
| 70.2459 | 149.813 | 82.397 | 79.408 | 153.374 |
| 70.4 | 159.176 | 82.397 | 83.1894 | 163.599 |
| 70.5659 | 155.431 | 82.397 | 85.08 | 173.824 |
| 70.72 | 147.94 | 82.397 | 85.08 | 163.599 |
| 70.8859 | 147.94 | 74.9064 | 71.8454 | 184.049 |
| 71.04 | 147.94 | 74.9064 | 81.2987 | 143.149 |
| 71.2059 | 147.94 | 78.6517 | 79.408 | 153.374 |
| 71.36 | 147.94 | 80.5243 | 79.408 | 153.374 |
| 71.5259 | 146.067 | 84.2697 | 71.8454 | 143.149 |
| 71.68 | 146.067 | 84.2697 | 83.1894 | 163.599 |
| 71.8459 | 151.685 | 84.2697 | 75.6267 | 173.824 |
| 72 | 147.94 | 84.2697 | 77.5174 | 173.824 |
| 72.1659 | 149.813 | 84.2697 | 79.408 | 184.049 |
| 72.32 | 147.94 | 84.2697 | 83.1894 | 184.049 |
| 72.4859 | 147.94 | 84.2697 | 81.2987 | 184.049 |
| 72.64 | 155.431 | 84.2697 | 81.2987 | 163.599 |
| 72.8059 | 159.176 | 84.2697 | 83.1894 | 184.049 |
| 72.96 | 147.94 | 84.2697 | 85.08 | 173.824 |
| 73.1259 | 144.195 | 84.2697 | 68.064 | 173.824 |
| 73.28 | 134.831 | 84.2697 | 71.8454 | 153.374 |
| 73.4459 | 146.067 | 84.2697 | 75.6267 | 184.049 |
| 73.6 | 149.813 | 84.2697 | 86.9707 | 153.374 |
| 73.7659 | 147.94 | 88.015 | 83.1894 | 184.049 |
| 73.92 | 151.685 | 89.8876 | 83.1894 | 184.049 |
| 74.0859 | 149.813 | 91.7603 | 83.1894 | 173.824 |
| 74.24 | 149.813 | 82.397 | 81.2987 | 143.149 |
| 74.4059 | 149.813 | 84.2697 | 79.408 | 184.049 |
| 74.56 | 147.94 | 84.2697 | 81.2987 | 163.599 |
| 74.7259 | 149.813 | 80.5243 | 79.408 | 143.149 |
| 74.88 | 147.94 | 82.397 | 79.408 | 153.374 |
| 75.0459 | 147.94 | 84.2697 | 81.2987 | 153.374 |
| 75.2 | 151.685 | 84.2697 | 83.1894 | 184.049 |
| 75.3659 | 157.303 | 84.2697 | 81.2987 | 153.374 |
| 75.52 | 157.303 | 84.2697 | 81.2987 | 184.049 |
| 75.6859 | 147.94 | 84.2697 | 86.9707 | 184.049 |
| 75.84 | 147.94 | 84.2697 | 83.1894 | 173.824 |
| 76.0059 | 149.813 | 84.2697 | 86.9707 | 163.599 |
| 76.16 | 147.94 | 84.2697 | 86.9707 | 143.149 |
| 76.3259 | 153.558 | 84.2697 | 83.1894 | 184.049 |
| 76.48 | 147.94 | 88.015 | 83.1894 | 184.049 |
| 76.6459 | 147.94 | 88.015 | 83.1894 | 153.374 |
| 76.8 | 151.685 | 76.779 | 85.08 | 173.824 |
| 76.9659 | 149.813 | 76.779 | 81.2987 | 184.049 |
| 77.12 | 147.94 | 78.6517 | 79.408 | 184.049 |
| 77.2859 | 151.685 | 84.2697 | 81.2987 | 184.049 |
| 77.44 | 146.067 | 84.2697 | 79.408 | 184.049 |
| 77.6059 | 144.195 | 84.2697 | 81.2987 | 163.599 |
| 77.76 | 146.067 | 86.1423 | 85.08 | 184.049 |
| 77.9259 | 146.067 | 84.2697 | 81.2987 | 184.049 |
| 78.08 | 146.067 | 84.2697 | 71.8454 | 184.049 |
| 78.2459 | 151.685 | 84.2697 | 79.408 | 184.049 |
| 78.4 | 149.813 | 84.2697 | 77.5174 | 184.049 |
| 78.5659 | 151.685 | 84.2697 | 75.6267 | 184.049 |
| 78.72 | 147.94 | 84.2697 | 69.9547 | 143.149 |
| 78.8859 | 157.303 | 84.2697 | 83.1894 | 173.824 |
| 79.04 | 159.176 | 84.2697 | 81.2987 | 184.049 |
| 79.2059 | 157.303 | 82.397 | 79.408 | 184.049 |
| 79.36 | 147.94 | 84.2697 | 79.408 | 184.049 |
| 79.5259 | 147.94 | 86.1423 | 86.9707 | 184.049 |
| 79.68 | 149.813 | 89.8876 | 77.5174 | 184.049 |
| 79.8459 | 147.94 | 93.633 | 68.064 | 194.274 |
| 80 | 134.831 | 93.633 | 68.064 | 184.049 |

**Table S2.** Data source for Fig. 2 (FTIR spectrum).

| Wavelength | Pebax/PEI/SiO2-20% | Pebax/PEI/SiO2-10% | Pebax/PEI/SiO2-5% | Pebax/PEI | SiO2 |
| --- | --- | --- | --- | --- | --- |
| 422.732 | 96.5785 | 96.6962 | 92.0443 | 96.0896 | 99.5798 |
| 439.88 | 93.469 | 91.066 | 97.6331 | 94.7132 | 91.1298 |
| 457.029 | 70.5869 | 84.4566 | 92.5307 | 91.8794 | 85.0607 |
| 474.177 | 58.0696 | 57.7743 | 82.4939 | 93.0129 | 75.9104 |
| 491.326 | 54.8007 | 41.1285 | 80.0232 | 89.2076 | 82.2596 |
| 508.475 | 71.7829 | 38.6806 | 84.9541 | 80.9492 | 90.0093 |
| 525.623 | 79.1976 | 46.8403 | 88.6513 | 72.124 | 95.9851 |
| 542.772 | 82.9449 | 75.2361 | 87.332 | 68.3187 | 96.6387 |
| 559.92 | 85.0178 | 81.8455 | 89.0555 | 72.5289 | 96.2185 |
| 594.217 | 85.0178 | 83.7222 | 89.0484 | 72.5289 | 96.2185 |
| 611.366 | 86.4529 | 83.7222 | 92.4167 | 71.2334 | 96.2185 |
| 628.514 | 90.9177 | 85.6806 | 92.1665 | 78.6822 | 96.2185 |
| 645.663 | 87.1705 | 90.7396 | 88.7911 | 78.6822 | 96.2185 |
| 662.812 | 92.9109 | 87.3125 | 97.834 | 74.0672 | 96.2185 |
| 679.96 | 96.3393 | 93.1059 | 94.8698 | 86.4548 | 96.2185 |
| 697.109 | 86.4529 | 96.7778 | 88.6161 | 78.1154 | 96.2185 |
| 714.257 | 82.1476 | 90.7396 | 88.8593 | 69.2093 | 96.2185 |
| 731.406 | 88.1272 | 82.4167 | 93.05 | 66.2136 | 96.2185 |
| 748.554 | 89.084 | 89.3524 | 90.9082 | 70.5047 | 96.2185 |
| 765.703 | 83.1043 | 89.3524 | 89.7533 | 75.4436 | 90.8964 |
| 782.851 | 93.7082 | 83.151 | 91.888 | 65.4849 | 85.3875 |
| 800 | 84.8584 | 93.2691 | 86.6212 | 77.8725 | 77.7311 |
| 817.149 | 79.5165 | 81.4375 | 88.7559 | 72.3669 | 73.296 |
| 834.297 | 75.5301 | 74.5017 | 84.8871 | 74.7959 | 77.0775 |
| 851.446 | 68.2748 | 70.2587 | 70.7383 | 72.4479 | 84.127 |
| 868.594 | 59.3452 | 64.3021 | 66.3761 | 56.0121 | 90.8964 |
| 885.743 | 74.7328 | 55.4896 | 80.6823 | 36.1757 | 95.1447 |
| 902.891 | 82.7854 | 73.5226 | 87.5869 | 47.2679 | 96.2185 |
| 920.04 | 84.9381 | 86.8229 | 86.9255 | 62.1654 | 95.4715 |
| 937.188 | 73.0585 | 87.7205 | 77.7933 | 64.9991 | 93.7908 |
| 954.337 | 63.9695 | 78.9896 | 74.6647 | 51.3971 | 89.916 |
| 971.486 | 58.0696 | 69.6059 | 71.2071 | 41.1146 | 83.4267 |
| 988.634 | 72.8193 | 62.9149 | 81.5658 | 36.8234 | 69.1877 |
| 1005.78 | 69.0721 | 70.3403 | 81.3978 | 53.6641 | 53.3147 |
| 1022.93 | 63.0925 | 71.4826 | 74.8973 | 54.3118 | 40.7563 |
| 1040.08 | 39.4928 | 59.5694 | 65.6829 | 49.373 | 29.9253 |
| 1074.38 | 25.62 | 41.0469 | 42.731 | 36.6615 | 23.9029 |
| 1091.53 | 20.4377 | 22.6875 | 25.6216 | 30.0224 | 21.0084 |
| 1108.67 | 17.6472 | 13.7934 | 17.3119 | 24.6787 | 19.0009 |
| 1125.82 | 19.7998 | 15.1806 | 14.0188 | 16.8252 | 19.8413 |
| 1142.97 | 19.7201 | 15.0174 | 15.1666 | 16.7442 | 21.5686 |
| 1160.12 | 17.408 | 13.7934 | 19.7685 | 16.7442 | 23.8562 |
| 1177.27 | 18.3647 | 13.7118 | 31.5252 | 16.7442 | 25.6303 |
| 1194.42 | 18.1255 | 13.7934 | 40.2391 | 18.0396 | 26.6573 |
| 1211.57 | 21.2349 | 14.9358 | 42.8673 | 17.7967 | 27.9645 |
| 1228.71 | 21.6336 | 13.7118 | 40.7255 | 22.5736 | 30.6256 |
| 1245.86 | 22.6701 | 13.7118 | 34.8007 | 18.2825 | 34.1737 |
| 1263.01 | 19.7998 | 15.099 | 31.9188 | 16.6632 | 39.8226 |
| 1280.56 | 21.2349 | 15.2622 | 29.2835 | 16.6632 | 44.5845 |
| 1297.71 | 19.7998 | 19.5052 | 36.4349 | 16.7442 | 49.6732 |
| 1314.86 | 31.9983 | 19.6684 | 49.8364 | 17.8777 | 53.3147 |
| 1332 | 44.3562 | 27.4201 | 60.2773 | 19.1731 | 55.5089 |
| 1349.15 | 49.9372 | 41.5365 | 55.9973 | 25.4074 | 57.6564 |
| 1366.3 | 31.3605 | 51.7361 | 43.2466 | 22.9785 | 59.2437 |
| 1366.3 | 20.4377 | 43.5764 | 28.5257 | 14.5581 | 60.2708 |
| 1383.45 | 29.9254 | 23.4219 | 29.838 | 15.1249 | 61.5313 |
| 1400.6 | 56.6344 | 29.0521 | 46.8581 | 20.4686 | 62.7918 |
| 1417.75 | 47.2265 | 53.3681 | 63.467 | 31.5607 | 63.6321 |
| 1434.9 | 50.894 | 59.7326 | 57.9535 | 25.5693 | 64.5658 |
| 1452.04 | 37.1009 | 52.0625 | 54.7426 | 23.1404 | 64.7059 |
| 1469.19 | 27.2146 | 47.5747 | 42.2386 | 15.2868 | 65.2194 |
| 1486.34 | 37.8982 | 33.4583 | 38.2053 | 16.7442 | 65.9197 |
| 1503.49 | 44.5157 | 33.9479 | 44.6165 | 19.6589 | 66.3866 |
| 1520.64 | 40.7684 | 45.3715 | 53.1659 | 22.3307 | 66.5266 |
| 1537.79 | 21.1552 | 53.2049 | 51.1064 | 17.3919 | 67.1802 |
| 1554.94 | 28.57 | 37.375 | 27.1711 | 15.5297 | 67.9272 |
| 1572.08 | 61.3384 | 19.9948 | 52.4974 | 18.9302 | 68.4874 |
| 1589.23 | 57.352 | 47.4931 | 67.1326 | 38.4427 | 67.8805 |
| 1606.38 | 33.4334 | 64.7917 | 62.2769 | 34.3945 | 67.6471 |
| 1623.53 | 17.0093 | 51.0833 | 51.9934 | 25.5693 | 68.254 |
| 1640.68 | 34.2307 | 44.7188 | 43.3547 | 16.9061 | 69.281 |
| 1640.68 | 59.5844 | 19.1788 | 30.0319 | 14.5581 | 69.7479 |
| 1657.83 | 62.4546 | 24.7274 | 24.9295 | 17.6348 | 69.888 |
| 1674.98 | 51.1332 | 52.2257 | 45.8971 | 36.2567 | 70.5882 |
| 1692.12 | 21.9525 | 65.3628 | 66.1245 | 35.528 | 70.5882 |
| 1709.27 | 45.1535 | 41.6181 | 56.5812 | 18.9302 | 70.1681 |
| 1726.42 | 81.7489 | 30.1128 | 24.422 | 29.5366 | 69.7479 |
| 1743.57 | 72.5802 | 66.0972 | 23.6783 | 62.975 | 69.7479 |
| 1760.72 | 88.5259 | 79.0712 | 77.8707 | 58.6839 | 69.7479 |
| 1777.87 | 93.7082 | 75.1545 | 78.2784 | 81.354 | 69.7479 |
| 1795.01 | 96.5785 | 93.5139 | 76.6301 | 92.5271 | 69.7479 |
| 1812.16 | 96.5785 | 95.5538 | 93.239 | 96.0896 | 69.7479 |
| 1829.31 | 96.5785 | 96.8594 | 96.3606 | 96.1705 | 70.1214 |
| 1846.46 | 95.1433 | 96.7778 | 97.6729 | 96.1705 | 70.1681 |
| 1863.61 | 95.1433 | 96.6962 | 97.5871 | 95.118 | 70.5882 |
| 1880.76 | 93.7082 | 95.309 | 97.5013 | 93.1748 | 70.9617 |
| 1897.91 | 93.7082 | 95.309 | 96.182 | 91.7175 | 71.0084 |
| 1915.05 | 93.7082 | 95.2274 | 96.0962 | 90.2601 | 71.1951 |
| 1932.2 | 93.7082 | 93.8403 | 96.1749 | 88.8837 | 71.4286 |
| 1949.35 | 93.7082 | 93.8403 | 94.6088 | 88.9647 | 71.4286 |
| 1966.5 | 94.0271 | 93.7587 | 94.6876 | 90.2601 | 71.8487 |
| 1983.65 | 95.1433 | 95.1458 | 96.0821 | 91.7175 | 72.0355 |
| 2000.8 | 95.1433 | 95.309 | 96.0786 | 93.1748 | 72.2689 |
| 2017.95 | 95.1433 | 95.309 | 96.1573 | 93.2558 | 72.6891 |
| 2035.09 | 95.0636 | 96.125 | 96.1538 | 93.1748 | 73.1092 |
| 2052.24 | 96.5785 | 96.7778 | 96.2325 | 93.1748 | 73.5294 |
| 2069.39 | 96.5785 | 96.8594 | 97.3803 | 93.1748 | 73.5294 |
| 2086.54 | 96.5785 | 96.8594 | 97.5413 | 93.1748 | 73.9496 |
| 2103.69 | 96.419 | 96.7778 | 97.62 | 93.1748 | 74.043 |
| 2120.84 | 95.1433 | 96.7778 | 97.6165 | 93.1748 | 74.183 |
| 2138.38 | 96.5785 | 96.7778 | 97.6128 | 93.1748 | 74.3697 |
| 2155.53 | 96.5785 | 96.8594 | 97.6093 | 93.2558 | 74.7899 |
| 2172.68 | 96.5785 | 96.7778 | 97.6058 | 94.6322 | 74.7899 |
| 2189.83 | 96.5785 | 96.8594 | 97.6023 | 94.7132 | 75.2101 |
| 2206.98 | 96.5785 | 96.7778 | 97.5987 | 94.6322 | 75.3968 |
| 2224.13 | 96.5785 | 96.7778 | 97.5952 | 93.5797 | 75.6303 |
| 2241.28 | 96.5785 | 96.7778 | 97.5917 | 93.1748 | 76.0037 |
| 2258.42 | 96.5785 | 96.8594 | 97.5882 | 93.1748 | 76.0504 |
| 2275.57 | 96.5785 | 96.7778 | 97.5846 | 93.2558 | 76.1905 |
| 2292.72 | 96.5785 | 96.8594 | 97.5811 | 93.2558 | 76.4706 |
| 2309.87 | 96.5785 | 96.7778 | 97.5776 | 94.6322 | 76.6573 |
| 2327.02 | 96.5785 | 96.8594 | 97.5741 | 94.6322 | 76.8908 |
| 2344.17 | 96.5785 | 96.7778 | 97.5705 | 94.7132 | 77.3109 |
| 2361.32 | 95.1433 | 96.7778 | 97.567 | 94.1464 | 77.451 |
| 2378.46 | 95.1433 | 96.8594 | 97.5635 | 93.2558 | 77.7311 |
| 2395.61 | 95.1433 | 96.8594 | 97.4777 | 93.2558 | 78.1513 |
| 2412.76 | 95.1433 | 96.7778 | 97.2275 | 93.1748 | 78.1513 |
| 2429.91 | 95.1433 | 95.4722 | 96.0726 | 93.1748 | 78.5714 |
| 2447.06 | 95.1433 | 95.309 | 95.9868 | 93.1748 | 78.9916 |
| 2464.21 | 95.1433 | 95.309 | 96.0655 | 92.932 | 78.9916 |
| 2481.36 | 94.1866 | 95.309 | 95.9798 | 91.7175 | 79.4118 |
| 2498.5 | 93.7082 | 95.2274 | 96.0585 | 91.6365 | 79.6452 |
| 2515.65 | 92.2731 | 93.6771 | 96.055 | 90.2601 | 79.8319 |
| 2532.8 | 92.2731 | 93.7587 | 95.9692 | 88.8028 | 80.2521 |
| 2549.95 | 93.7082 | 93.8403 | 96.0479 | 88.8028 | 80.6723 |
| 2567.1 | 92.2731 | 93.8403 | 95.9621 | 88.8028 | 80.719 |
| 2584.25 | 92.2731 | 93.6771 | 94.4783 | 88.8028 | 81.0924 |
| 2601.4 | 90.7583 | 91.5556 | 94.4748 | 88.7218 | 81.0924 |
| 2618.54 | 87.9678 | 89.6788 | 94.5535 | 86.6167 | 81.5126 |
| 2635.69 | 86.3732 | 87.7205 | 94.4677 | 80.8682 | 81.9328 |
| 2652.84 | 82.7854 | 85.8438 | 94.4642 | 75.6055 | 82.3529 |
| 2669.99 | 79.2774 | 83.4774 | 94.3784 | 70.3428 | 82.7731 |
| 2687.14 | 78.9584 | 81.9271 | 91.5788 | 60.789 | 82.7731 |
| 2704.29 | 74.8923 | 80.3767 | 90.0949 | 50.8303 | 83.1933 |
| 2738.58 | 68.3545 | 74.5833 | 87.1272 | 40.8717 | 83.6134 |
| 2755.73 | 61.0195 | 66.5868 | 86.9592 | 26.5409 | 84.267 |
| 2772.88 | 44.7549 | 57.6111 | 85.4754 | 15.4488 | 84.8739 |
| 2790.03 | 16.9296 | 32.4792 | 81.8533 | 16.4203 | 84.7339 |
| 2807.18 | 16.6904 | 18.8524 | 76.093 | 13.9104 | 85.0607 |
| 2824.33 | 18.3647 | 22.6875 | 69.9215 | 15.1249 | 85.901 |
| 2841.48 | 19.7998 | 24.1563 | 57.9932 | 13.7485 | 86.3212 |
| 2858.62 | 18.3647 | 40.6389 | 25.7517 | 15.7726 | 85.8543 |
| 2875.77 | 21.2349 | 61.691 | 28.1332 | 29.2127 | 85.8077 |
| 2892.92 | 38.3766 | 80.7031 | 31.5015 | 53.1783 | 85.7143 |
| 2910.07 | 68.9126 | 83.3958 | 31.4979 | 70.0189 | 85.7143 |
| 2927.22 | 79.9949 | 80.5399 | 30.343 | 71.2334 | 85.7143 |
| 2944.37 | 82.1476 | 72.8698 | 39.3859 | 62.5702 | 86.1345 |
| 2961.52 | 76.3274 | 69.0347 | 58.462 | 45.6486 | 86.1811 |
| 2978.66 | 67.2383 | 75.1545 | 76.469 | 54.9595 | 86.5546 |
| 2996.21 | 67.4775 | 79.1528 | 86.2519 | 64.1085 | 86.1345 |
| 3013.36 | 73.5369 | 84.4566 | 87.6464 | 72.7717 | 86.1345 |
| 3030.51 | 80.553 | 85.0278 | 83.202 | 77.0629 | 86.1345 |
| 3047.66 | 83.5827 | 84.7014 | 75.7146 | 77.9535 | 86.2278 |
| 3064.81 | 85.0178 | 81.8455 | 75.9578 | 71.3144 | 86.5546 |
| 3081.95 | 83.6624 | 77.6024 | 81.8755 | 65.242 | 86.5546 |
| 3099.1 | 78.3206 | 75.9705 | 86.5597 | 62.3273 | 86.5546 |
| 3133.4 | 74.8923 | 75.7257 | 89.9245 | 61.0319 | 86.1811 |
| 3167.7 | 74.7328 | 70.0139 | 86.7101 | 53.9879 | 86.1345 |
| 3184.85 | 72.2612 | 56.224 | 83.9926 | 40.9526 | 86.1345 |
| 3201.99 | 64.687 | 38.9253 | 83.7424 | 25.0026 | 86.0878 |
| 3219.14 | 51.4521 | 18.934 | 82.1763 | 18.5254 | 85.7143 |
| 3236.29 | 23.2282 | 18.6076 | 76.7449 | 15.3678 | 85.2941 |
| 3253.44 | 16.8499 | 41.2917 | 65.5568 | 16.6632 | 84.8739 |
| 3270.59 | 19.0025 | 59.5694 | 47.625 | 21.683 | 84.4538 |
| 3287.74 | 47.7846 | 67.6476 | 21.716 | 38.0379 | 83.7535 |
| 3304.89 | 61.7371 | 73.033 | 21.4657 | 49.5349 | 83.0999 |
| 3322.03 | 67.7964 | 77.5208 | 51.3975 | 58.441 | 82.2596 |
| 3339.18 | 73.9355 | 80.3767 | 68.9933 | 67.59 | 81.9328 |
| 3356.33 | 78.5598 | 82.2535 | 76.2268 | 73.9053 | 81.5126 |
| 3373.48 | 81.9881 | 83.3958 | 79.5951 | 77.0629 | 81.9328 |
| 3390.63 | 83.503 | 82.0903 | 84.197 | 76.9819 | 81.9328 |
| 3407.78 | 83.5827 | 81.9271 | 86.9074 | 75.6055 | 82.1662 |
| 3424.93 | 83.5827 | 83.0694 | 88.302 | 74.1481 | 82.2129 |
| 3459.22 | 83.5827 | 83.3958 | 88.4594 | 75.4436 | 82.493 |
| 3476.37 | 83.5827 | 83.4774 | 88.3736 | 76.2532 | 83.24 |
| 3493.52 | 83.5827 | 84.9462 | 88.2879 | 76.9819 | 84.0336 |
| 3579.26 | 85.0178 | 85.0278 | 89.915 | 76.9819 | 84.7339 |
| 3596.41 | 85.0178 | 86.4149 | 91.1451 | 78.5202 | 85.4342 |
| 3613.56 | 85.6556 | 87.8021 | 91.3883 | 81.273 | 86.0411 |
| 3630.71 | 87.7286 | 89.434 | 92.8651 | 84.5116 | 86.7414 |
| 3647.86 | 90.2002 | 90.9028 | 94.2596 | 87.4264 | 87.4883 |
| 3665 | 92.2731 | 92.3715 | 95.7364 | 90.1792 | 88.1419 |
| 3682.15 | 93.7082 | 94.0035 | 96.3908 | 91.9604 | 88.8422 |
| 3699.3 | 95.1433 | 95.7986 | 97.2919 | 95.2799 | 89.5892 |
| 3716.45 | 96.4987 | 96.7778 | 97.2062 | 96.1705 | 90.3361 |
| 3733.6 | 96.5785 | 96.7778 | 97.2026 | 96.1705 | 90.7563 |
| 3750.75 | 96.5785 | 96.8594 | 97.2813 | 96.0896 | 91.3632 |
| 3767.9 | 96.5785 | 96.8594 | 97.2778 | 96.0896 | 92.0168 |
| 3785.04 | 96.5785 | 96.8594 | 97.2743 | 96.1705 | 92.437 |
| 3802.19 | 96.5785 | 96.7778 | 97.2708 | 96.1705 | 92.8571 |
| 3819.34 | 96.5785 | 96.8594 | 97.2672 | 96.1705 | 92.9505 |
| 3836.49 | 96.5785 | 96.7778 | 97.2637 | 96.1705 | 93.5107 |
| 3854.04 | 96.5785 | 96.8594 | 97.2601 | 96.1705 | 93.8842 |
| 3871.19 | 96.5785 | 96.7778 | 97.2566 | 96.1705 | 94.4444 |
| 3888.33 | 96.5785 | 96.8594 | 97.2531 | 96.1705 | 94.5378 |
| 3905.48 | 96.5785 | 96.7778 | 97.2495 | 96.1705 | 94.5378 |
| 3922.63 | 96.5785 | 96.7778 | 97.246 | 96.1705 | 94.958 |
| 3939.78 | 96.5785 | 96.8594 | 97.2425 | 96.1705 | 95.3782 |
| 3956.93 | 96.5785 | 96.8594 | 97.239 | 96.1705 | 95.7983 |
| 3974.08 | 96.5785 | 96.8594 | 97.2354 | 96.1705 | 96.1251 |
| 3991.23 | 96.5785 | 96.7778 | 97.3141 | 96.0896 | 96.2185 |


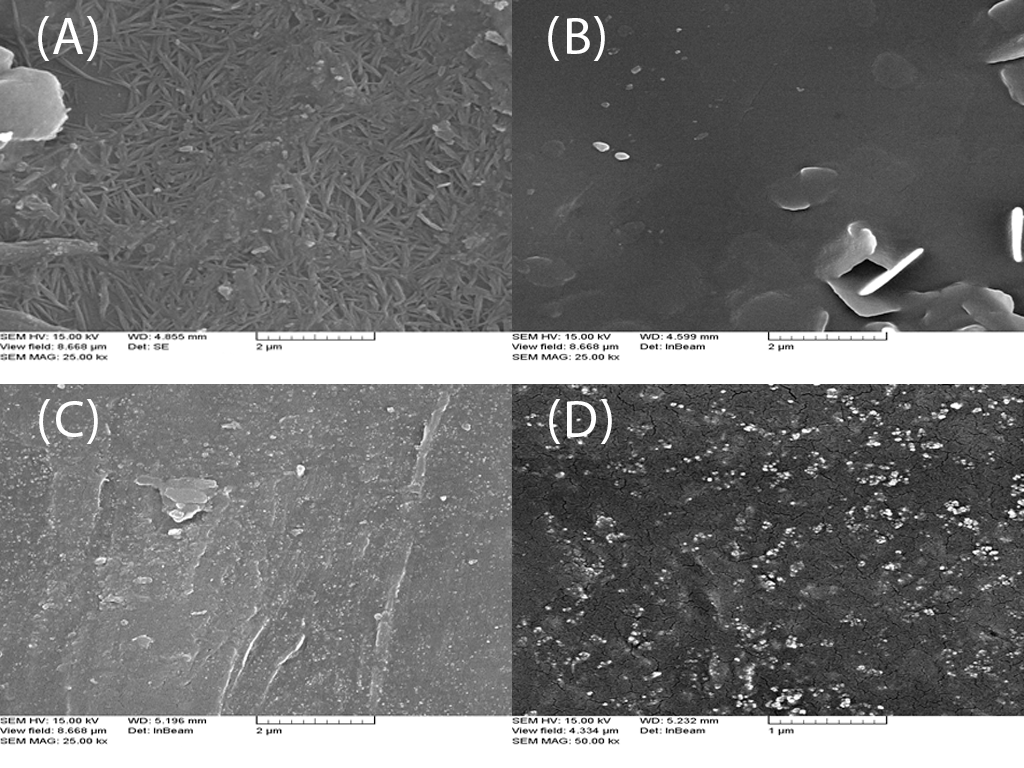


**Fig. 3.** Data source for SEM images of fabricated membranes, A) Pebax/PEI, B) Pebax/PEI/${SiO}_{2}$-5%, C) Pebax/PEI/${SiO}_{2}$-10% and D) Pebax/PEI/${SiO}_{2}$-20%.

**Table S3.** Data source for Fig. 4 (The effect of nanoparticle loading on permeability in various feed gas pressure for $N_{2}$, $O_{2}$, ${CH}_{4}$ and ${CO}_{2}$).

|  | **Gas type** | N2 | | | |
| --- | --- | --- | --- | --- | --- |
|  | **Pressure** | 2 bar | 4 bar | 6 bar | 8 bar |
| **Membrane** | Pebax/PEI | 0.392377 | 0.156951 | 0.190583 | 0.213004 |
|  | Pebax/PEI/SiO2-5% | 1.5583 | 0.840807 | 0.605381 | 0.706278 |
|  | Pebax/PEI/SiO2-10% | 2.00673 | 1.6704 | 1.00897 | 1.43498 |
|  | Pebax/PEI/SiO2-20% | 2.10762 | 1.86099 | 1.49103 | 1.86099 |
|  | **Gas type** | O2 | | | |
|  | **Pressure** | 2 bar | 4 bar | 6 bar | 8 bar |
| **Membrane** | Pebax/PEI | 1.00216 | 0.829741 | 0.689655 | 0.711207 |
|  | Pebax/PEI/SiO2-5% | 1.64871 | 0.959052 | 0.711207 | 0.980603 |
|  | Pebax/PEI/SiO2-10% | 1.95043 | 1.78879 | 1.02371 | 1.55172 |
|  | Pebax/PEI/SiO2-20% | 2.13362 | 1.89655 | 1.64871 | 1.98276 |
|  | **Gas type** | CH4 | | | |
|  | **Pressure** | 2 bar | 4 bar | 6 bar | 8 bar |
| **Membrane** | Pebax/PEI | 1.84821 | 1.73661 | 1.65848 | 1.94866 |
|  | Pebax/PEI/SiO2-5% | 2.49554 | 2.27232 | 2.00446 | 2.54018 |
|  | Pebax/PEI/SiO2-10% | 2.97545 | 2.80804 | 2.12723 | 2.74107 |
|  | Pebax/PEI/SiO2-20% | 3.2433 | 2.94196 | 2.76339 | 3.02009 |
|  | **Gas type** | CO2 | | | |
|  | **Pressure** | 2 bar | 4 bar | 6 bar | 8 bar |
| **Membrane** | Pebax/PEI | 11.0991 | 7.97414 | 6.03448 | 9.80603 |
|  | Pebax/PEI/SiO2-5% | 17.6724 | 15.194 | 14.1164 | 16.4871 |
|  | Pebax/PEI/SiO2-10% | 19.8276 | 17.8879 | 15.0862 | 19.9353 |
|  | Pebax/PEI/SiO2-20% | 20.5819 | 19.8276 | 16.5948 | 20.0431 |

**Table S4.** Data source for Table 1 (The kinetics diameter and condensability).

| Condensability (K) | Kinetic diameter (Å) | Gas |
| --- | --- | --- |
| 195 | 3.3 | $\mathbf{CO}_{\mathbf{2}}$ |
| 107 | 3.46 | $\mathbf{O}_{\mathbf{2}}$ |
| 71 | 3.64 | $\mathbf{N}_{\mathbf{2}}$ |
| 149 | 3.8 | $\mathbf{CH}_{\mathbf{4}}$ |

**Table S5.** Data source for Table 2 (The selectivity of prepared membranes).

| **Pressure** | **Gas type** | **Membrane type** | | | |
| --- | --- | --- | --- | --- | --- |
|  |  | **Pebax/PEI** | **Pebax/PEI-5%** | **Pebax/PEI-10%** | **Pebax/PEI-20%** |
| **2 Bar** | $\mathrm{CH}_{4}$ | 6.05324 | 7.081594 | 6.663732 | 6.345975 |
|  | $O_{2}$ | 11.07518 | 10.71893 | 10.16576 | 9.646469 |
|  | $N_{2}$ | 28.28683 | 11.34082 | 9.880552 | 9.76547 |
| **4 Bar** | $\mathrm{CH}_{4}$ | 4.591785 | 6.686558 | 6.370244 | 6.739589 |
|  | $O_{2}$ | 9.610396 | 15.84273 | 10 | 10.45456 |
|  | $N_{2}$ | 50.80656 | 18.07073 | 10.70875 | 10.65433 |
| **6 Bar** | $\mathrm{CH}_{4}$ | 3.638561 | 7.042495 | 7.091946 | 6.005233 |
|  | $O_{2}$ | 8.749998 | 19.84851 | 14.73679 | 10.06532 |
|  | $N_{2}$ | 31.66326 | 23.31821 | 14.95208 | 11.12976 |
| **8 Bar** | $\mathrm{CH}_{4}$ | 5.032191 | 6.490524 | 7.272817 | 6.63659 |
|  | $O_{2}$ | 13.78787 | 16.81323 | 12.84723 | 10.10869 |
|  | $N_{2}$ | 46.03683 | 23.34364 | 13.89239 | 10.77013 |
